# High gene flow in the silverlip pearl oyster *Pinctada maxima* between inshore and offshore sites near Eighty Mile Beach in Western Australia

**DOI:** 10.7717/peerj.13323

**Published:** 2022-05-31

**Authors:** Luke Thomas, Karen J. Miller

**Affiliations:** 1 Oceans Institute, Oceans Graduate School, The University of Western Australia, Crawley, Australia; 2 Indian Ocean Marine Research Centre, Australian Institute of Marine Science, Crawley, Australia

**Keywords:** Silverlip Peal Oyster, Genetic connectivity, Eighty Mile Beach, DArT Seq

## Abstract

An understanding of stock recruitment dynamics in fisheries is fundamental to successful management. *Pinctada maxima* is a bivalve mollusc widely distributed throughout the Indo-Pacific and is the main species targeted for cultured pearl and pearl shell production in Australia. Pearl production in Australia relies heavily on wild-caught individuals, the majority of which come from the Eighty Mile Beach region near Broome in Western Australia. In this study, we used a genotyping by sequencing approach to explore fine-scale patterns of genetic connectivity among inshore shallow and offshore deep populations of *P. maxima* near Eighty Mile Beach. Our results revealed high-levels of gene flow among inshore and offshore sites and no differences in genetic diversity between depths. Global estimates of genetic differentiation were low (*F*_ST_ = 0.006) but significantly different from zero, and pairwise estimates of genetic differentiation among sites were significant in only 3% of comparisons. Moreover, Bayesian clustering detected no separation of inshore and offshore sample sites, and instead showed all samples to be admixed among sites, locations and depths. Despite an absence of any clear spatial clustering among sites, we identified a significant pattern of isolation by distance. In a dynamic environment like Eighty Mile Beach, genetic structure can change from year-to-year and successive dispersal and recruitment events over generations likely act to homogenize the population. Although we cannot rule out the null hypothesis of panmixia, our data indicate high levels of dispersal and connectivity among inshore and offshore fishing grounds.

## Introduction

Sustainable fisheries management relies heavily on an accurate understanding of stock-recruitment dynamics and patterns of dispersal in order to set biologically relevant spatial boundaries and harvest limits (Leis, Herwerden & Patterson, 2011; Palumbi, 2003). Most fisheries-targeted invertebrate marine species have a bipartite life cycle which consists of a relatively sessile adult stage and a dispersing larval stage, with the larval stage the primary vector for dispersal from natal sites. Larval dispersal is governed by the complex interplay between larval biology (*e.g*. swimming capacity, larval duration) and oceanographic process that act to restrict or promote dispersal and facilitate connectivity (Kinlan, Gaines & Lester, 2005; Levin, 2006; Weersing & Toonen, 2009). As a result, most marine populations exist along a continuum of connectivity, from highly connected populations that regularly exchange larvae, to isolated populations cut-off from the broader metapopulation (Waples & Gaggiotti, 2006). The strength of connections among local populations have broad implications for population maintenance and replenishment following intensive harvesting or severe environmental disturbances (Bernhardt & Leslie, 2013; Harrison et al., 2020).

**Figure 1:**
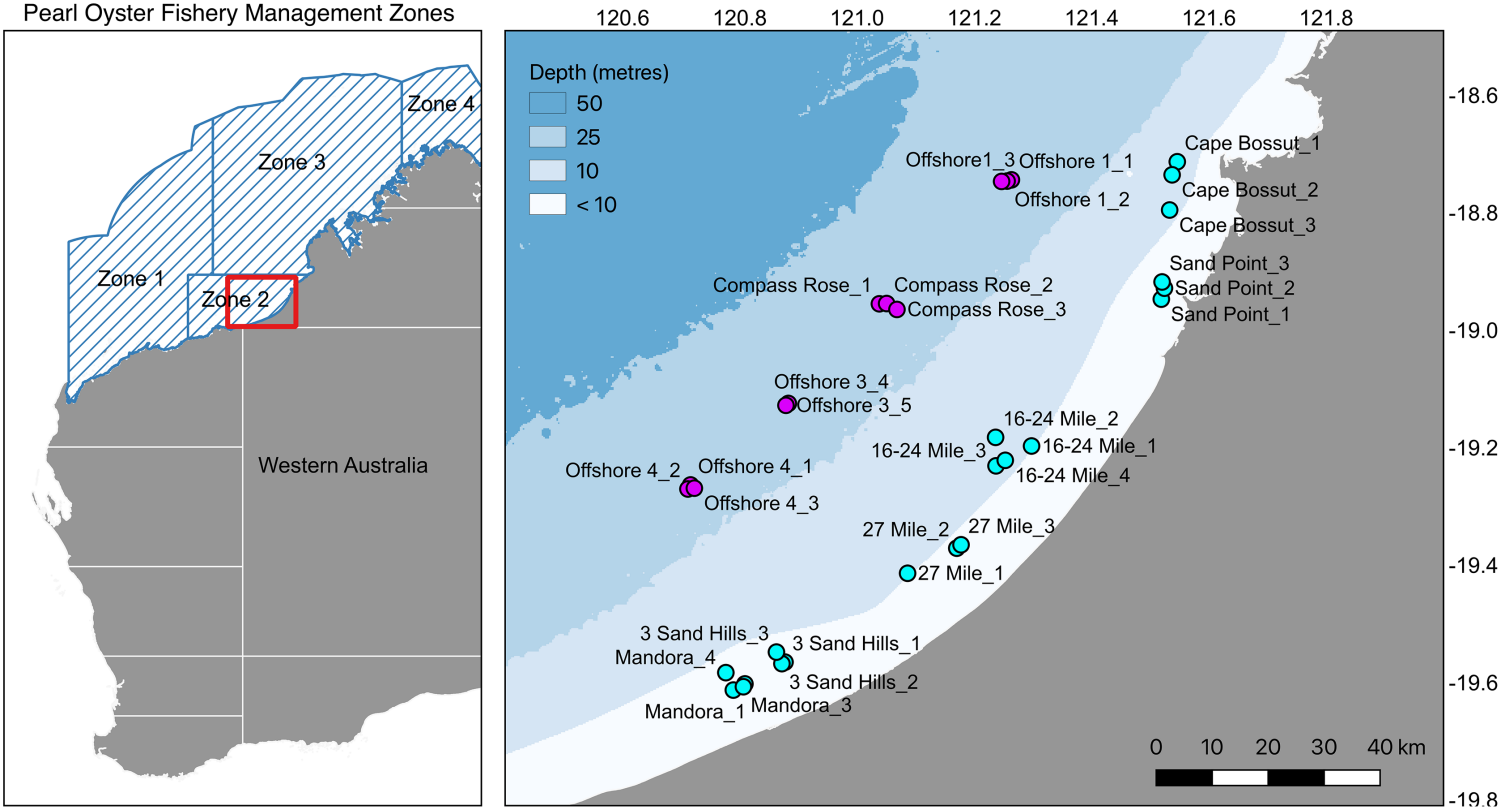
Map of sampling locations. Pearl Oyster Fishery Zones in Western Australia and locations of sample sites in Zone 2 along Eighty Mile Beach near Broome in Western Australia. A total of 715 silverlip pearl oysters were collected from 33 sites in the Eighty Mile Beach area (Fishery Zone 2) by commercial divers on surface-supplied air operating from Industry fishing vessels during neap tides. Samples were collected along two depth contours representing inshore shallow (8–15 m, cyan) and offshore deep (35 m, magenta).

The silverlip pearl oyster, *Pinctada maxima*, is a bivalve mollusc widely distributed throughout the Indo-Pacific and is the main species targeted for cultured pearl and pearl shell production in the region. *Pinctada maxima* is the largest of the *Pinctada* species used for pearl production (Rose & Baker, 1994), and the only species used for culturing pearls in Australia. Pearl production in Australia relies heavily on wild-caught individuals, and the Australian fishery represents the last remaining wild-capture pearl oyster fishery in the world. Each year, approximately 500,000 individuals are harvested from the wild, with the majority of fishing focused along Eighty Mile Beach in Western Australia (Zone 2-Fisheries Report 2016, Fisheries Research and Development Corporation, 2016; Fig. 1). Wild oysters are collected by divers on surface-supplied air and transported to commercial aquaculture farms for pearl production. Commercial diving for pearl oysters is restricted to depths less than 35 m and occurs predominantly in nearshore habitats in 8–15 m depth.

*Pinctada maxima*, along with other *Pinctada* species, is a broadcast spawning hermaphrodite with a pelagic larval duration of approximately 3–4 weeks (Gervis & Sims, 1992). This moderate larval duration in this group of commercially important oysters translates to gene flow within-regions, but seemingly insufficient to prevent regionally structured genetic populations (Arnaud-Haond et al., 2008; Lal et al., 2017; Lemer & Planes, 2014; Reisser et al., 2020; Rozenfeld et al., 2008). Nonetheless, amidst a backdrop of gene flow within regions, local populations may be structured at fine-spatial scales owing to local environment forces and the stochastic nature of recruitment in highly-fecund invertebrates (Lemer & Planes, 2014; Reisser et al., 2020). For example, complex circulation patterns associated with closed lagoon habitats in French Polynesia act to restrict dispersal and generate fine-scale genetic patchiness in the black-lipped pearl oyster *P. margaritifera* that does not reflect an expected pattern of isolation by distance (Lemer & Planes, 2014).

There is only limited evidence of connectivity in *P. maxima* between inshore and offshore sites along Eighty Mile Beach. At the regional scale, *P. maxima* populations are characterized by strong genetic structure between Indonesia and Australia, and show a strong decline in genetic diversity with latitude (Benzie & Smith-Keune, 2006; Johnson & Joll, 1992; Lind et al., 2007). Within Australia, strong genetic subdivisions occur between populations in Northern Territory, Queensland, and Western Australia (Benzie & Smith-Keune, 2006; Johnson & Joll, 1992) indicating an absence of panmixia at the National scale. Western Australian *P. maxima* populations are considered a single stock, with the exception of the most southerly populations within the Exmouth Gulf (Benzie & Smith-Keune, 2006; Johnson & Joll, 1992). In the Eighty Mile Beach area, oceanographic models predict that large tidal currents (up to 8 m tides) move larvae back and forth across the shelf and connect inshore and offshore fishing grounds (Condie & Hart, 2006). An absence of any genetic structure in the Eighty Mile Beach region supports extensive larval exchange between shallow and deep populations (Benzie & Smith-Keune, 2006); however, previous genetic studies have been limited in both sampling design and resolution of genetic markers to unravel any fine-scale patterns in population structure that may occur in this region.

Here, we re-visit the genetic structure in *P. maxima* from inshore and offshore sites near Eighty Mile Beach in Western Australia. We used a genotyping by sequencing approach and an extensive nested sampling design to explore patterns of connectivity between deep and shallow populations in a region of high economic and cultural importance. Based on previous genetic and oceanographic studies that included samples from this region, we hypothesize that gene flow is high between deep and shallow sites, and that *P. maxima* populations are not genetically structured across the Eighty Mile Beach region.

## Methods

### Sample collection

A total of 715 silverlip pearl oysters were collected from 33 sites in the Eighty Mile Beach area (Fishery Zone 2) by commercial divers on surface-supplied air operating from Industry fishing vessels during neap tides on two sampling trips; shallow inshore samples (<10 m) were collected in May/June 2018, and offshore deep samples (>30 m) collected in July 2019 (Fig. 1). Sampling was conducted using a spatially replicated, hierarchical design including two depths, up to five locations within each depth (separated by ~20 kms), and with oysters collected at up to five replicate sites within each location (separated by 1–5 km). At each sample site, approximately 20 individual adult oysters were collected within as small an area as possible (typically the first 200–250 m of a standard drift dive; Table S1). All samples collected from inshore (8–15 m; *n* = 467) were from regular fishing grounds. Samples collected offshore (~35 m; *n* = 248) included one deep fishing ground (Compass Rose) and three other areas where moderate densities of pearl oysters had been recorded during oyster habitat towed video and multibeam sonar surveys (Whalan et al., 2021). Tissue samples of the adductor muscle were taken from each oyster and immediately preserved in 100% AR grade ethanol for DNA extraction. Maximum length measurements were recorded for each shell before tissue biopsies were taken under Fisheries Exemptions P12018 and P12019.

### Reduced representation sequencing

We generated reduced representation libraries using Pstl-HpaII restriction enzymes at Diversity Arrays Technology (DArT). DArTseq is conceptually similar to RADseq methods and uses enzymes to fragment DNA for sequencing. In this case, two enzymes were used to increase the number of fragments for SNP calling. The PstI-compatible adapter included Illumina flowcell attachment sequence, sequencing primer sequence and sample barcode region. Reverse adapter contained flowcell attachment region and HpaII-compatible overhang sequence. Libraries are amplified in 30 rounds of PCR using the following reaction conditions: PCR conditions consisted of an initial denaturation at 94 °C for 1 min followed by 30 cycles of 94 °C for 20 s, 58 °C for 30 s and 72 °C for 45 s, with a final extension step at 72 °C for 7 min. Equimolar amounts of amplification product are sequenced on an Illumina Hiseq2500 (single end 77 cycles). FASTQ files were processed for poor quality (>Q20) and identical sequences are collapsed into “fastqcall files”, which are groomed using DArT’s proprietary algorithm that corrects low quality bases from singleton tags using collapsed tags with multiple members as a template. These files are used in the secondary pipeline for DArT PL’s proprietary SNP calling algorithms (DArTsoft14). All tags from all libraries were clustered using DArT PL’s C++ algorithm at the threshold distance of three, followed by parsing of the clusters into separate SNP loci using a range of technical parameters, especially the balance of read counts for the allelic pairs. Additional selection criteria were added to the algorithm based on analysis of approximately 1,000 controlled cross populations. Testing for Mendelian distribution of alleles in these populations facilitated selection of technical parameters discriminating true allelic variants from paralogous sequences and contaminating sequences that has been achieved through training DArTsoft14 proprietary software’s capacity to perform this “filtering” of viral and/or bacterial sequences based on analysis of thousands of control crosses in large diversity of organisms. Equimolar amounts of 744 libraries were sequenced across nine lanes on an Illumina Hiseq2500 (single end 77 cycles). Raw *fastq* files were processed for poor quality (>Q20) and identical reads. These filtered reads were used in the secondary pipeline for DArT PL’s proprietary SNP calling algorithms (DArTsoft14).

### Quality filtering

All statistical analyses were implemented using R software, version 3.0.1 unless noted (R Core Team, 2015). We filtered the raw DArT genotype matrix consisting of 11,280 binary SNPs for minor allele frequencies (0.05) call rate (0.90 loci and individual) and coverage (10×) using *dartR* (Gruber et al., 2018). We also removed sample sites with less than 10 individuals, and locations without replicate sample sites. Like other methods for reduced representation sequencing, DArTseq datasets can be affected by allele dropout, which leads to an apparent heterozygosity deficit in the population. Many of the population-level analyses rely on loci being in HWE (Falush, Stephens & Pritchard, 2003; Nei, 1973), so it is crucial that datasets are screened thoroughly for deviations from HWE. To this end, we removed the remaining loci out of HWE using *gl.filter.hwe* function after adjusting for multiple comparisons using the Bonferroni method. Any locus that showed departures from HWE at more than one site was removed from the dataset. Finally, we removed any *F*_ST_ outlier loci that could confound interpretations of gene flow with *outflank* (Foll & Gaggiotti, 2008) and fsthet (Flanagan & Jones, 2017). *Outflank* analyses were carried out using a 5% left and right trim for the null distribution of *F*_ST_, minimum heterozygosity for loci of 0.1% and a 5% false discovery rate (*qvalue*). Outliers were identified with *fsthet* using the *fthetboot* function and based on an alpha value of 0.05 and 1,000 reps. Loci identified as *F*_ST_ outliers under either approach were removed from the dataset prior to exploring patterns of population genetic structure.

### Genetic diversity and population differentiation

To test for significant differences in genetic diversity between inshore and offshore samples, we calculated expected heterozygosity for each sample site using *poppr* (Kamvar, Tabima & Grünwald, 2014) and then used ANOVA (*aov*) to test for the significance of depth on heterozygosity. Overall levels of inbreeding (*F*_IS_) were calculated using *hierfstat* (Goudet, 2005) and assessed for significance using 95% confidence intervals (CI) calculated by bootstrapping over loci. We also calculated observed and expected heterozygosity across all loci using the *summary* function on a *genind* object, and used the Bartlett test of homogeneity of variance as implemented in *stats* to test for significant heterozygosity deficits, which are common in marine bivalves (Johnson & Joll, 1992). Global estimates of genetic differentiation (Nei, 1972) were calculated in *mmod* (Winter, 2012) and departures from panmixis among sites were tested by bootstrapping (nboots = 10,000) over loci. Pairwise estimates of genetic differentiation among sample sites were calculated in *stampp* (Pembleton, Cogan & Forster, 2013) and we adjusted for multiple comparisons with *p.adjust* at the FDR 0.05 significance level.

### Spatial clustering and genetic connectivity

To test for significant genetic divergence among inshore and offshore sites, we performed a hierarchical analysis of molecular variance (AMOVA) in *pegas* and tested for significance using the *randtest* function. Samples were nested by depth, location within depth, and site within location. Evidence for spatial genetic structure among deep and shallow sites was further explored using individual-based principle components analysis with *ade4* (Dray & Dufour, 2007) using Euclidean distance matrices, and by constructing a neighbour-joining dendrogram based of Nei’s genetic distance in *poppr* with bootstrap support for tree nodes based on 10,000 bootstrap replicates. To identify the optimal number of genetic clusters in our dataset, we used model-based Bayesian clustering in *structure* (Pritchard, Stephens & Donnelly, 2000) and Bayesian Information Criteria (BIC) using discriminant analysis of principle components (DAPC) as implemented in *adegenet* (Jombart, 2008). *Structure* analyses were carried out using population information (loc prior), correlated allele frequencies, a burn-in of 200,000 MCMC iterations and 500,000 iterations for each run. The number of K ranged from 1 to 10 (number of sampled sites), with five replicate analyses for each K value. The appropriate number of K was identified by comparing the likelihood of the data for different values of K and using the ∆K method in *structure harvester* (Earl & vonHoldt, 2012). Results were then averaged using *clummp* (Jakobsson & Rosenberg, 2007) to minimize variance across iterations, before graphics were generated. To determine if the observed pattern of genetic differentiation reflected one of isolation by distance, we used Mantel’s Test in *ade4* and calculated pairwise geographic distances, calculated as the shortest path between two given sites using *geosphere*. Mantel tests were applied to the entire data set, and for offshore and inshore locations separately. We bootstrapped across tests for significance using the *mantel.randtest* function in *ade4*. Finally, we used multi-locus spatial autocorrelation analysis to identify the scale of spatial genetic structure and calculated autocorrelation coefficients (r) among sites for each species using *PopGenRepor*t (Adamack & Gruber, 2014).

## Results

**Figure 2:**
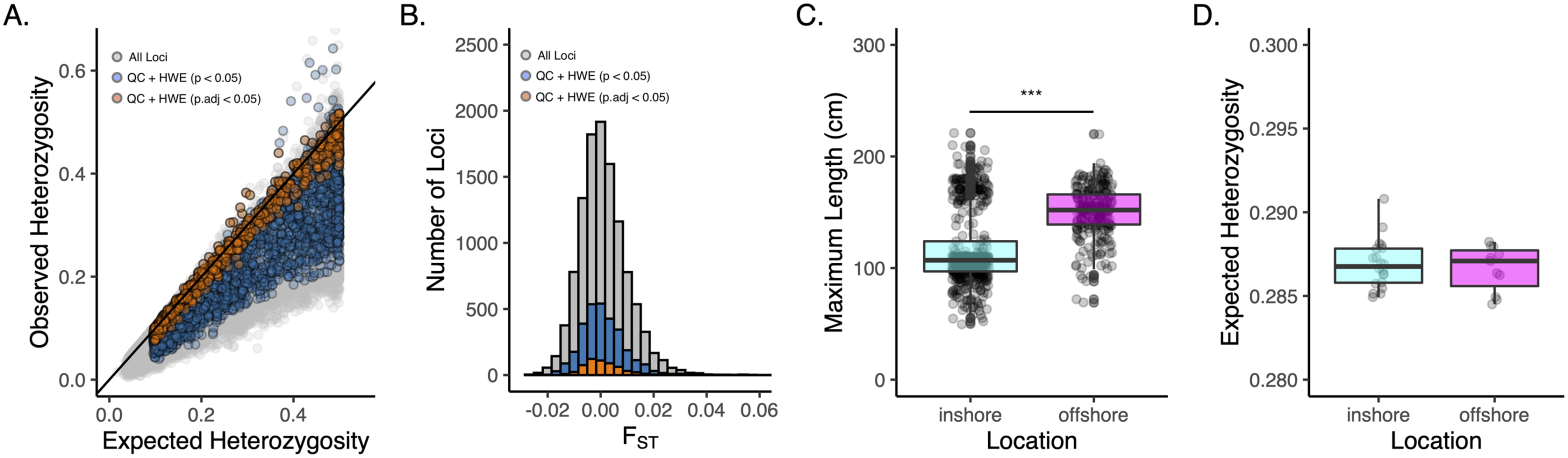
Genetic diversity and overall structure across sample sites. (A) Scatter plot of observed v. expected heterozygosity per locus in *Pinctada maxima* based on the original DArT dataset (grey-11,280 loci), post filtering (blue-2,986 loci), and following additional HWE filtering step using an unadjusted *P*-value of 0.05 (orange-559 loci). Black solid line indicates the 1:1 relationship. Loci are colour coded by stage of data QC; (B) Histogram of FST across all loci from original DArT dataset (grey-11,280 loci), post QC (blue-2,986 loci), and following additional HWE filtering step using an unadjusted *p*-value of 0.05 (orange-559 loci); (C) Boxplots of size structure (maximum length in centimetres) and (D) expected heterozygosity for inshore (cyan) and offshore (magenta) sites based on 2,986 SNPs that passed data QC. ***denotes significance at the *p* < 0.001 threshold.

Approximately 2M reads per sample (+/− 13,496 SE) were used for variant calling in DArTsoft14, which returned 11,280 binary SNPs (Table 1). After filtering for call rate, minor allele frequency, coverage, and HWE, we were left with 2,986 loci called across 664 individual oysters collected from 31 sites from 10 locations and two depths (Table S1; Figs. S1–S3). Our *F*_ST_-based outlier scans revealed no locus to be a significant outlier and so we used all 2,986 loci for analyses of population genetic structure (Fig. S4). Despite our filtering criteria that removed loci out of HWE (*p*.adj < 0.05), we observed a strong and significant (*p* < 0.001) heterozygote deficit in the total population, with only 18% of all loci that passed QC (559 SNPs) to be in strict HWE before adjusting for multiple comparisons at the 0.05 significance level (Fig. 2A-orange points). Downstream analyses were carried out using both datasets, which produced consistent results, unless otherwise noted. These datasets and accompanying scripts and raw *fastq* files are available *via* the Open Science Framework (https://osf.io/v9u38/).

**Table 1:** Sampling information and descriptive statistics.

*N*	Reads per sample	DArTseq	SNP_QC_	*F*_ST_	*H*_E_	*H*o	*F*_IS_
664	2,004,482 (+/− 13,517)	11,280	2,986	0.006*	0.288	0.227	0.211*

**Note:**

Sampling information and descriptive statistics: (N) number of sampled pearl oysters; (reads per sample) number of reads used to generate genotype data; (DArTseq) number of single nucleotide polymorphisms (SNPs) that passed through the DArTSoft pipeline; (SNPQC) number of SNPs that passed quality control filters; (FST) Wright’s Fixation index; (HE) Expected and (HE) Observed heterozygosity across all sites; (FIS) inbreeding coefficient. Significance is denoted as an asterisk (*).

**Figure 3:**
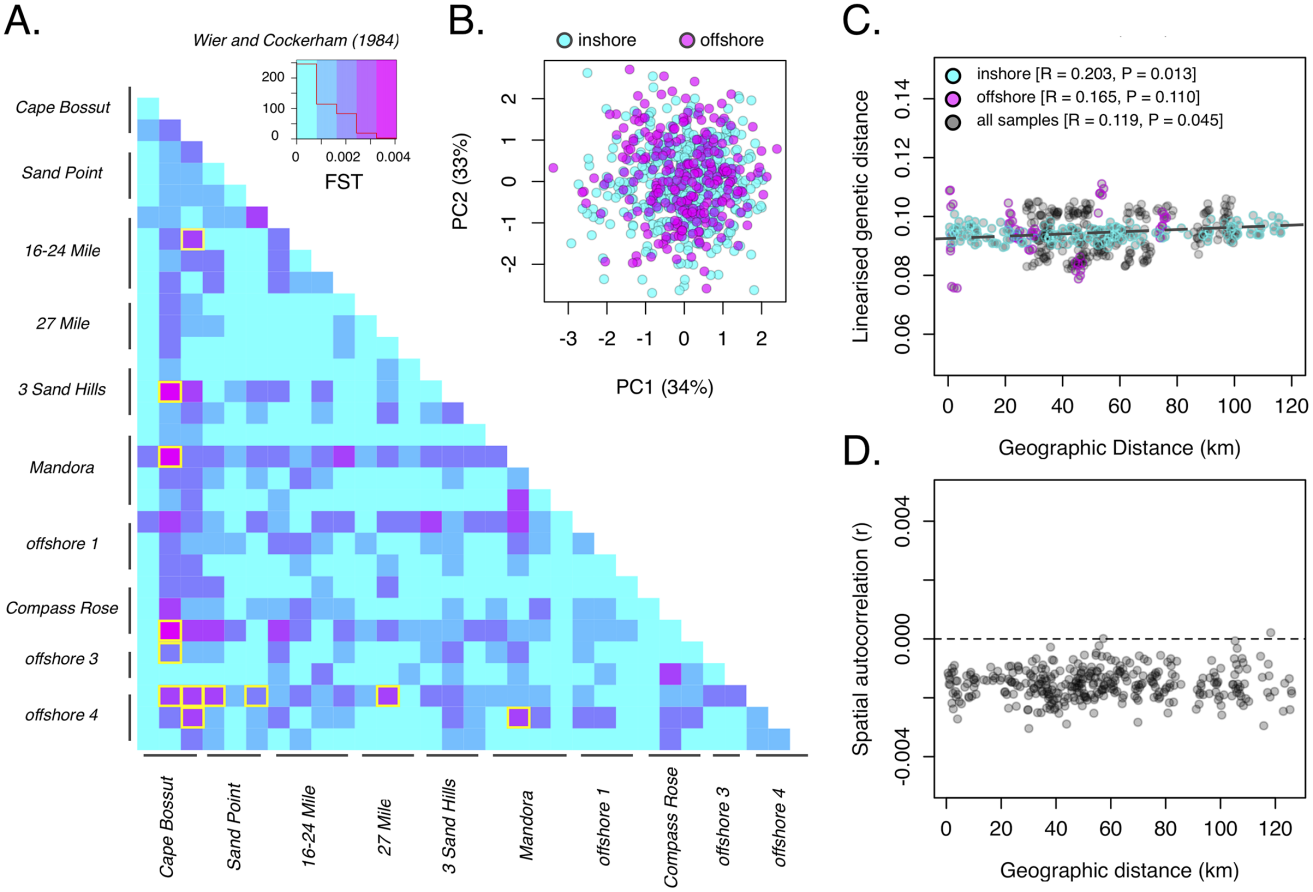
Population genetic structure of *P. maxima* near Eighty Mile Beach in northwest Australia. (A) Pairwise estimates of genetic differentiation (FST, Weir & Cockerham, 1984) among sites based on 2,986 loci. Red trace in legend is a histogram for all pairwise values and significant pairwise comparisons (*n* = 12) are outlined in yellow; (B) scatterplot of the first two principal components with sample sites coloured by depth (cyan-inshore, magenta-offshore, black-all); (C) scatterplot of geographic distance and linearised genetic distance (isolation by distance) for all sites (black) and with inshore (cyan) and offshore (magenta) sites exclusively; (D) scatterplot of genetic correlation coefficients among samples with increasing geographic distance.

Consistent with the strong heterozygote deficit in the population, global inbreeding coefficients were high (F_IS_ = 0.211) and significantly different from zero at all sample sites (Table S2). Although oysters from offshore sites were generally larger in size than inshore sites (Fig. 2C; *P* < 0.001), we did not detect any differences in genetic diversity (as *H*_E_) between depths (Fig. 2D). A global estimate of genetic differentiation (Nei, 1972) was low but significantly different from zero (*F*_ST_ = 0.006, CI [0.005–0.007]), ruling out the null hypothesis of panmixia. Despite a significant global *F*_ST_ value, only 3% of pairwise comparisons among sites (*n* = 12) were significant after correcting for multiple comparisons (Fig. 3A). More than half of these significant comparisons involved a Cape Bossut site (*n* = 8), which was the most northerly of our inshore sample locations, or an Offshore four site (*n* = 7), which was the most southerly of our offshore sample sites. None of these comparisons, however, remained significant when restricting our analyses to the subset of loci in strict HWE. Similarly, hierarchical analysis of molecular variance (AMOVA) indicated that a small but significant portion of genetic variation was attributed to differences between sites within location (0.074%; *P* = 0.01) and depth (0.026%; *P* = 0.01, Table S2); however, depth did not account for a significant portion of the genetic variation when restricting our analyses to the subset of loci in strict HWE (Table S3).

Consistent with low levels of differentiation under the AMOVA framework, there were no obvious spatial clustering of samples by depth based on PCA (Fig. 3B), all samples were admixed across sites, locations, and depth. Population-level clustering based on Nei’s (1972) genetic distance revealed a more complex relationship among sites and showed some evidence of divergence in most offshore sites, but without strong bootstrap support across all nodes (<50%; Fig. S5). Bayesian clustering in *structure* revealed that under the *∆K* method, the most likely number of genetic clusters in our dataset was *K* = 2 (Fig. S6), but admixture plots showed all samples to be completely admixed among sites, locations and depths (Fig. S7). These patterns were confirmed by the BIC method that identified K = 1 as the optimal number of clusters in the data, with no spatial clustering of sites using DAPC (Fig. S8). Despite an absence of any clear spatial clustering among sites, we identified a weak and marginally significant pattern of isolation by distance (Mantel’s R = 0.119, *P* = 0.045; Fig. 3C). This pattern remained significant when focussing exclusively on inshore (Mantel’s R = 0.203, *P* = 0.013) but not offshore (Mantel’s R = 0.165, *P* = 0.110) sites. Finally, spatial autocorrelation analysis showed no significant positive autocorrelation (greater than random genetic similarity) at most spatial scales in the dataset (Fig. 3D), reconfirming the lack of spatial genetic structure among our sample sites.

## Discussion

The overall pattern of genetic structure in the silverlip pearl oyster *Pinctada maxima* from inshore and offshore sites near Eighty Mile Beach in Western Australia reflects one of high gene flow. Levels of genetic differentiation among sites were low and Bayesian clustering analyses indicated that all samples formed a single genetic population with no obvious separation of inshore and offshore sites. These patterns of genetic homogeneity across out study site are consistent with *Pinctada* species from other regions across similar spatial scales (Arnaud-Haond et al., 2008; Lal et al., 2016; Lemer & Planes, 2014; Takeuchi et al., 2020), and of dispersing marine invertebrates more generally, that are often characterized by strong regional genetic structure layered on top of high (but stochastic) gene flow among local populations (Weersing & Toonen, 2009).

The patterns of high gene flow identified in this study are not surprising considering the limited spatial extent of our study, the moderate larval duration of the species (Rose & Baker, 1994), and the large tidal currents of the region (Condie & Hart, 2006). The continental shelf in northwest Australia extends 100s of km, resulting in some of the largest tides in the world (Lowe et al., 2015). These tides drive strong cross-shelf currents that can carry *P. maxima* larvae tens of kilometres (Condie & Hart, 2006). Particle dispersal modelling and spat surveys indicate that inshore populations along Eighty Mile Beach are largely self-seeding, with spawning and recruitment concentrated along the 8–15 m contour, and with intermittent dispersal offshore (Condie & Hart, 2006). Despite these findings, there is a long-standing hypothesis in the pearl industry that offshore populations represent a brood stock of larvae for inshore areas. We were not able to identify any clear directionality to gene flow between depths; however, our data clearly show that there is sufficient cross-shelf dispersal to homogenize the genetic structure of *P. maxima* in the region. Oysters collected from offshore sites were larger in size than inshore oysters, but these differences may reflect our different sampling strategies rather than a result of the gauntlet fishing strategy of the industry. Pearl oyster collection inshore was conducted during normal commercial diving operations, and it is possible that divers preferentially selected smaller shells for our study that were not size for culture and retained larger animals to be sold to the boat for profit. The collections offshore were only for scientific purposes and therefore size was not confounded by fishing preferences.

Our initial screening for deviations from HWE based on an adjusted significance threshold revealed a strong heterozygote deficit across thousands of loci. Heterozygote deficits are common in marine bivalves (David et al., 1997; Mcgoldrick & Hedgecock, 1997), and have been previously reported in *P. maxima* in northwest Australia using allozymes and microsatellite markers (Benzie & Smith-Keune, 2006; Johnson & Joll, 1992; Lind et al., 2007). An excess of homozygotes is generally attributed to the Wahlund effect, where genetically discrete populations are sampled as one (Pusack et al., 2014), or due to allele drop out, where an allele at a locus fails to amplify. Although some pairwise comparisons were significant, the low levels of genetic differentiation among sample sites rule out the influence of the Wahlund effect as the driving force behind the heterozygosity deficits observed here. Further our smallest spatial scale of sampling (hundreds of metres) was likely well below the spatial scale of genetic populations. If we rule out the possibility of Wahlund effect or allele drop out resulting in this signal, then the heterozygote deficits observed in *P. maxima* near Eighty Mile Beach could be the result of inbreeding and assortative mating driven by subtle differences in the timing of spawning of individuals and/or patchiness in larval recruitment (Mcgoldrick & Hedgecock, 1997). However, this is not known to occur in *Pinctada*, and considering the strength of the inbreeding signal, it is possible that allele dropout is causing the observed deficits in heterozygosity observed across most of the loci.

Estimates of genetic differentiation among sample sites showed that approximately 3% of pairwise comparisons were significant, most of which included a Cape Bossut site or an Offshore 4 site. It may be the case that complex currents and oceanographic features that form around the Cape act to entrap larvae and isolate nearby populations. Pearl oyster habitat in the Cape Bossut area is likely seeded by larvae to the north of Eighty Mile Beach region, possibly explaining the patterns of genetic differentiation observed here (Condie & Hart, 2006). These patterns of differentiation, however, were not consistent across replicate sites within our Cape Bossut sampling location, with one of the more northern Cape Bossut sites showing a lack of any significant genetic differentiation with other sampling sites. Interestingly, many of the significant pair-wise tests also included sites from Offshore 4–and localised current patterns would seem unlikely to explain this pattern. Notably, all significant pairwise values, however, became non-significant when we recalculated *F*_*ST*_ based on a small subset of loci in strict HWE, cautioning interpretations of the significant genetic differentiation observed here. Nevertheless, populations of highly fecund marine invertebrates often reflect a ‘sweepstakes’ chance of reproductive success, where a small group of individuals can account for a large proportion of the successful recruits to a given area (Christie et al., 2010). This results in patterns of chaotic genetic patchiness, where one generation of genetic patches cannot predict the following generation (Johnson & Black, 1982, 1984). It is likely that in a dynamic environment like Eighty Mile Beach, population structure is fluid and can change from year to year, and although significant differentiation arises in the population, successive dispersal and recruitment events over generations homogenize the population. As a result, the genetic patches are not discrete populations, but rather a small part of a larger genetic mosaic that fluctuates through time. Only through long-term temporal monitoring can we begin to unravel the complex interplay between larval biology, ocean currents, and the space-time continuum.

## Supplemental Information

10.7717/peerj.13323/supp-1 Supplemental Information 1 Supplemental Figures and Tables.

## Additional Information and Declarations

### DNA Deposition

The following information was supplied regarding the deposition of DNA sequences: The raw sequences are available at SRA: PRJNA720157

### Data Availability

The following information was supplied regarding data availability: The Raw DArTseq genotype matrix is available in the Supplemental File.
