# Supplementary material for: High gene flow in the silverlip pearl oyster Pinctada maxima between inshore and offshore sites near Eighty Mile Beach in Western Australia
Source: PeerJ. 2022 May 31;10:e13323. doi: 10.7717/peerj.13323 (PMC9165592; doi:10.7717/peerj.13323)
Supplement: Supplemental Information 1 [file peerj-10-13323-s001.docx]

**Electronic Supplementary Material**

**High gene flow in the silver-lipped pearl oyster *Pinctada maxima* between inshore and offshore sites near Eighty-Mile Beach in northwest Australia**

L Thomas^1,2^ and KJ Miller^1^

^1^ Australian Institute of Marine Science, Indian Ocean Marine Research Centre, Crawley, 6009

^2^ Oceans Graduate School & The UWA Oceans Institute, The University of Western Australian, Crawley, 6009

Table S1 Sample size, expected and observed heterozygosity at each sample site.

| **Sample site** | ***N*** | ***H_O_*** | ***H_E_*** | ***F_IS_ (CI)*** |
| --- | --- | --- | --- | --- |
| 16-24 Mile_1 | 22 | 0.22736049 | 0.27878425 | 0.1979-0.2182 |
| 16-24 Mile_2 | 22 | 0.22724942 | 0.27992939 | 0.2017-0.2225 |
| 16-24 Mile_3 | 21 | 0.22493169 | 0.27814418 | 0.2063-0.2254 |
| 16-24 Mile_4 | 21 | 0.23346193 | 0.27987738 | 0.1791-0.1996 |
| 27 Mile_1 | 21 | 0.22384077 | 0.27880619 | 0.2120-0.2307 |
| 27 Mile_2 | 22 | 0.22633764 | 0.27982959 | 0.2043-0.2242 |
| 27 Mile_3 | 22 | 0.22878398 | 0.28057731 | 0.1948-0.2177 |
| 3 Sand Hills_1 | 21 | 0.2282767 | 0.27874897 | 0.1931-0.2147 |
| 3 Sand Hills_2 | 20 | 0.22549452 | 0.27760312 | 0.2044-0.2241 |
| 3 Sand Hills_3 | 22 | 0.23047983 | 0.28110825 | 0.1924-0.2128 |
| Cape Bossut_1 | 20 | 0.22576787 | 0.27956586 | 0.2060-0.2277 |
| Cape Bossut_2 | 20 | 0.23921837 | 0.28075936 | 0.1625-0.1822 |
| Cape Bossut_3 | 20 | 0.22786874 | 0.28166084 | 0.2033-0.2263 |
| Mandora_1 | 19 | 0.22751167 | 0.27948519 | 0.1986-0.2239 |
| Mandora_2 | 20 | 0.22671074 | 0.2804435 | 0.2068-0.2273 |
| Mandora_3 | 21 | 0.23750075 | 0.28369026 | 0.1784-0.1997 |
| Mandora_4 | 21 | 0.22405707 | 0.27816242 | 0.2063-0.2296 |
| Sand Point_1 | 22 | 0.23071765 | 0.27961027 | 0.1877-0.2064 |
| Sand Point_2 | 22 | 0.2292439 | 0.27962662 | 0.1919-0.2135 |
| Sand Point_3 | 20 | 0.22582366 | 0.28060735 | 0.2112-0.2294 |
| Compass Rose_1 | 17 | 0.22519645 | 0.27616684 | 0.2022-0.2259 |
| Compass Rose _2 | 16 | 0.22873031 | 0.27825872 | 0.1995-0.2266 |
| Compass Rose_3 | 19 | 0.225504 | 0.27861613 | 0.2071-0.2279 |
| offshore_30m_1_1 | 31 | 0.22618571 | 0.28321974 | 0.2089-0.2264 |
| offshore_30m_1_2 | 30 | 0.2252338 | 0.28004946 | 0.2041-0.2221 |
| offshore_30m_1_3 | 30 | 0.22700628 | 0.28216278 | 0.2037-0.2234 |
| offshore_30m_3_4 | 17 | 0.22593652 | 0.27856988 | 0.2076-0.2310 |
| offshore_30m_3_5 | 16 | 0.22442661 | 0.27865127 | 0.2147-0.2392 |
| offshore_30m_4_1 | 26 | 0.22511105 | 0.28057475 | 0.2090-0.2276 |
| offshore_30m_4_2 | 21 | 0.23047259 | 0.28117837 | 0.1905-0.2130 |
| offshore_30m_4_3 | 22 | 0.22270065 | 0.27784158 | 0.2121-0.2344 |

Table S2 Hierarchical analysis of molecular variance (AMOVA) based on 2,986 loci.

| AMOVA 2,986 SNPs | Sigma | % variation | PhiST | p.val |
| --- | --- | --- | --- | --- |
| Variation between Depth | 0.175 | 0.026 | 0.000 | 0.010 |
| Variation between Location within Depth | -0.110 | -0.016 | 0.000 | 0.860 |
| Variation between Site within Location | 0.501 | 0.074 | 0.001 | 0.010 |
| Variation between Samples within Site | 104.226 | 15.346 | 0.154 | 0.010 |
| Variation within Samples | 574.368 | 84.570 | 0.154 | 0.010 |

Table S3 Hierarchical analysis of molecular variance (AMOVA) based on 599 loci in strict HWE.

| AMOVA 599 SNPs | Sigma | % variation | PhiST | p.val |
| --- | --- | --- | --- | --- |
| Variation between Depth | 0.010 | 0.007 | 0.000 | 0.380 |
| Variation between Location within Depth | -0.012 | -0.008 | 0.000 | 0.630 |
| Variation between Site within Location | 0.127 | 0.089 | 0.001 | 0.030 |
| Variation between Samples within Site | 5.024 | 3.532 | 0.035 | 0.010 |
| Variation within Samples | 137.069 | 96.380 | 0.036 | 0.010 |

Figure S1 Histogram of call rate by individual before (above) and after (below) filtering at the 0.90 threshold.

Figure S2 Histogram of call rate by locus before (above) and after (below) filtering at the 0.90 threshold.

Figure S3 Histogram of coverage per locus before (left) and after (right) filtering.

Figure S4 Expected heterozygosity (He) as a function of *F*_ST_. No locus was identified as an *F*_ST_ outlier by OUTFLANK or FSTHET.

Figure S5. Unrooted neighbouring-joining tree of the 31 sample sites along Eighty-Mile Beach. Bootstrap values were low (<50%) across all nodes and so not shown. Sample sites are coloured by depth.

Figure S6. Results from Structure Harvester identifying K=2 as the most likely number of clusters in the dataset.


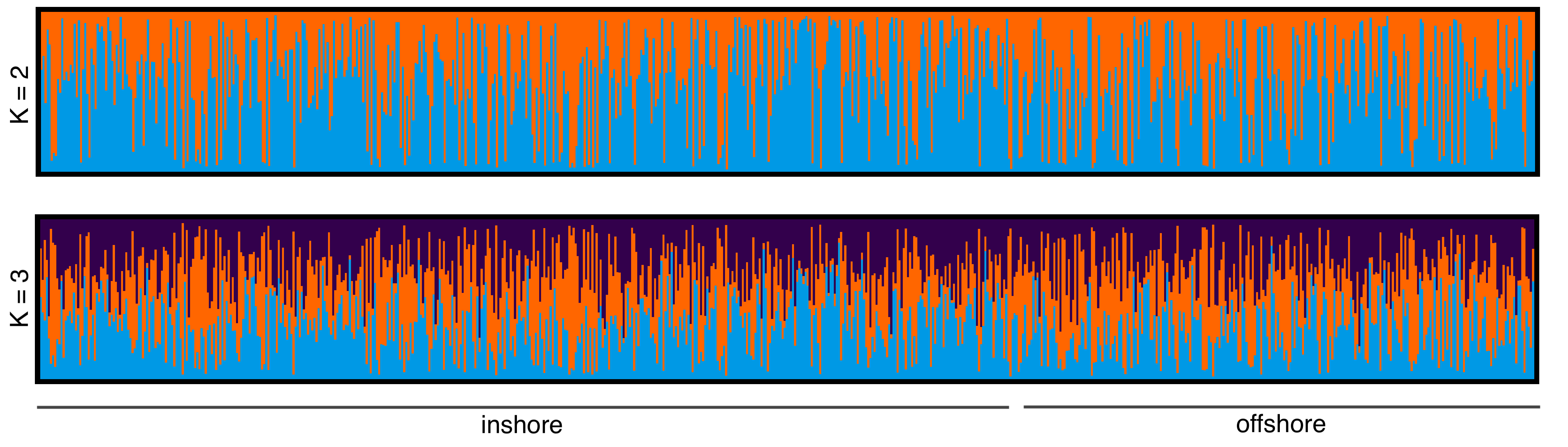


Figure S7 Results from Bayesian admixture in Structure at K=2 and K=3.

Figure S8 Number of genetic clusters in the dataset under the BIC method.
